# Cycloid psychosis: revisiting the concept through a case report

**DOI:** 10.1192/j.eurpsy.2025.2197

**Published:** 2025-08-26

**Authors:** C. Alves e Cunha, R. M. Cabral, P. Pires, I. Santos, F. Cunha, N. Cunha

**Affiliations:** 1 Departamento de Psiquiatria e Saúde Mental, ULS Viseu Dão-Lafões, Viseu, Portugal

## Abstract

**Introduction:**

The term cycloid psychosis was first introduced by Karl Kleist in 1926 to describe cases that did not meet the typical presentations of schizophrenia or manic-depressive illness. The concept was later developed by Karl Leonard, who proposed three distinct types of cycloid psychoses. Perris and Brockington established the first operational diagnostic criteria for the condition. Cycloid psychosis is characterized by its acute onset, brief duration, polymorphous and shifting symptomatology, a tendency for periodic recurrence, and full inter-episode recovery, with no residual functional impairment following the episodes. Despite its distinct characteristics, cycloid psychosis is not included in the current international psychiatric classification systems.

**Objectives:**

We aim to present a case report and conduct a literature review.

**Methods:**

A narrative review of the literature was conducted, with data collected from the PubMed database. Only English-language studies published in peer-reviewed journals were included in the selection.

**Results:**

Case: A 35-year-old female patient has been presenting, over the last seven years, with episodes characterized by a sudden onset of agitation, acute anxiety (described by the patient as ‘an inexplicable sense of fear’), and frequently accompanied by persecutory delusions and auditory commanding hallucinations. Upon observation, the patient typically presents with confusion and perplexity and exhibits a lack of recollection of some previous events. Following the initiation of antipsychotic treatment, a complete recovery is always observed within a few days, with no residual symptoms remaining. In the most recent episode, the patient drove for a few hours to a different city from the one where she lives and had to ask for help from a fire department, as she felt lost and confused and had no memory of the previous events. According to reports from her family, the patient exhibited disorganized behavior, increased irritability, and a reduction in sleep duration during the week preceding the episode. The patient later acknowledged discontinuation of antipsychotic medication. These episodes occurred without any prior substance use, cognitive decline, or underlying medical conditions. The patient had no previous psychiatric complaints or family history of psychiatric disorders. Although the patient was a smoker, there was no history of substance or alcohol abuse. A comprehensive evaluation, including laboratory tests, imaging studies (head CT and brain MRI) and electroencephalography (EEG), revealed no abnormal findings. Considering the range of symptoms and characteristics observed in this clinical case, the patient meets Perris’s criteria for cycloid psychosis.

**Conclusions:**

Our case report highlights that cycloid psychosis exhibits a distinctive symptom pattern and clinical outcome, which can support its validity as a nosological construct distinguishable from other disorders in classification systems.

**Disclosure of Interest:**

None Declared

